# Polymorphisms in Cha o 1 and Cha o 2, major allergens of Japanese cypress (*Chamaecyparis obtusa*) pollen from a restricted region in Japan

**DOI:** 10.1371/journal.pone.0261327

**Published:** 2021-12-28

**Authors:** Minoru Tateno, Ayaka Enami, Koji Fujinami, Hisashi Ohta

**Affiliations:** Torii Pharmaceutical Co. Ltd., Tokyo, Japan; Austrian Federal Research Centre for Forests BFW, AUSTRIA

## Abstract

Japanese cedar pollinosis is a major seasonal allergy in Japan, and Japanese cypress pollinosis is a growing concern because the cypress pollen season follows the cedar pollen season and cross-reactivity among allergens occurs between these closely related species. Allergens purified from pollen under unspecified collecting conditions can potentially heterogenous allergens profiles and batch to batch variability, and amino acid sequence variants in allergens possibly exist among trees. Polymorphisms have not been investigated for the cypress pollen major allergens, Cha o 1 and Cha o 2. Our aim was to examine the homogeneity of allergen amino acid sequences. DNA sequences of Cha o 1 and Cha o 2 from pollen collected from Chiba and Ibaraki prefectures and from needles of 47 plus trees located at seed orchards in Chiba Prefecture were examined by amplicon sequencing and amino acid substitutions were deduced. Sequence analysis of the pollen samples revealed that eight and seven residues of Cha o 2 were polymorphic, respectively. Thirteen residues in Cha o 2, including those residues identified in pollen, were deduced to be polymorphic for the plus trees. Cha o 2 expressed by the 47 plus trees included amino acid differences when compared with that of isoallergen Cha o 2.0101. No substitution was deduced in Cha o 1 for pollen taken from the two prefectures. One conservative amino acid substitution was deduced in Cha o 1 for the plus trees. Of the 47 plus trees examined, 38 were deduced to express only the isoallergen Cha o 1.0101 isoform, whereas eight trees were heterozygous and a single tree was homozygous for the non-synonymous mutation, which indicates relative uniformity of Cha o 1. Cha o 2 was found to be a heterogeneous allergen which suggests that studies using pollen from different trees may not give the same results.

## Introduction

Cupressaceae is a widely distributed conifer family whose pollen causes allergic diseases [1]. In the Mediterranean region, Arizona cypress (*Cupressus arizonica*) and Mediterranean cypress (*Cupressus sempervirens*) release pollen that cause allergic reactions, and a major allergen from Mediterranean cypress is the most common allergen among 75 allergens found in Italy [2]. In North America, Mountain cedar (*Juniperius ashei*) is a major source of pollen of Cupressus that causes allergic reactions [3]. Pollen from the Japanese cedar (*Cryptomeria japonica*) is responsible for the most serious seasonal allergic disease in Japan. The number of cedar pollinosis patients has increased over the years and was 26.5% of the Japanese population in 2008 [4, 5]. Japanese cypress (*Chamaecyparis obtusa*) belongs to the same family as the Japanese cedar and its pollen also causes allergic reactions. Japanese cedar and Japanese cypress, endemic species to Japan, have been planted extensively and occupy 18% and 10% of forestland in Japan, respectively [6]. Pollination of the cedar begins in February and continues through to April, whereas Japanese cypress pollen diffuses into the air between March and May [7]. Major allergens of Japanese cedar, Cry j 1 and Cry j 2, have been isolated, and genetic and biochemical analysis has revealed that these allergens are cell wall degrading enzymes [8–11].

Allergen immunotherapy (AIT) is the only treatment that modifies allergic diseases [5]. AIT for 3 years is beneficial even after treatment is discontinued. Conversely, treatment with widely used medicines such as antihistamines or oral corticosteroids provide only limited short-term relief from allergic diseases. The mechanism of AIT is not fully understood; however, this treatment is postulated to induce blocking IgG4 and modification of T-helper 1/T-helper 2 cell balance [12]. For treating Japanese cedar pollinosis (JCP), a Japanese cedar pollen extract containing both Cry j 1 and Cry j 2 in both solution and sublingual tablet forms are available for use in subcutaneous and sublingual immunotherapies [13]. Homologous molecules Cha o 1 and Cha o 2 were isolated as major allergens from the Japanese cypress pollen [14–16]. The high sequence identities between Cry j 1 and Cha o 1, and Cry j 2 and Cha o 2 suggests the existence of cross reactivity between these allergens against the corresponding cedar-pollen-specific IgE. A significant proportion of pollen-specific IgE from patients with seasonal allergy rhinitis has been shown to react with cedar and cypress extracts [17]. Thus, the Japanese cedar pollen extract may reduce symptoms caused by both the cedar and cypress pollen. Nonetheless it remains controversial whether the cedar pollen extract is sufficient to treat symptoms caused by the cypress pollen [18, 19].

Examining the cross allergenicity of IgE from JCP patients toward homologous allergens should benefit from using allergens that are uniform and composed of defined sequences because amino acid substitutions in the B cell epitope can affect binding avidity between the antibody and allergen [20, 21]. Cry j 1 and Cry j 2 have variations in their amino acid sequences [20, 22]; however, no survey has examined possible variations in the amino acid sequences of Cha o 1 and Cha o 2 allergens. Recombinant technology is often used to obtain uniform allergen molecules with a defined sequence. However, soluble recombinant Cry j 1, Cry j 2, Cha o 1 and Cha o 2 and their homologous allergens from related species are currently unavailable. An alternative approach involves identifying trees that express allergen species of a defined sequence. For Japanese cedar and Japanese cypress, those candidate trees may be found from mother trees in seed orchards used for plantation because Japanese cedar and Japanese cypress are cultivars. Modern plantation of cedar and cypress in Japan involves selecting trees with superior growth and pathogen-resistant properties. These trees, termed plus trees, have been expanded by grafting and are maintained in orchards. Progenies from their cross are used for plantations [23].

In this report, the homogeneity of the primary sequences of Cha o 1 and Cha o 2 was examined by surveying possible polymorphisms in Cha o 1 and Cha o 2 allergen DNA from pollen collected in Chiba Prefecture, which is located east of Tokyo area. Cha o 1 and Cha o 2 genomic DNA sequences from 47 plus trees at seed orchards governed by Chiba Prefecture were also examined. For comparison, we surveyed polymorphisms in Cha o 1 and Cha o 2 from pollen collected in a different prefecture but the same breeding district of Japan, Ibaraki Prefecture, which is just north of Chiba Prefecture. We found that the Cha o 1 amino acid sequence was essentially uniform with only one amino acid substitution identified. Conversely, Cha o 2 was found to have a variety of amino acid substitutions with significant frequencies. Composition and substitution frequencies in Cha o 2 deduced from pollen collected from Chiba Prefecture was partially different when compared with that collected from Ibaraki Prefecture. We raise the possibility that different source materials include different frequencies of Cha o 2 isoforms. We also discuss the possibility that Cha o 2 bearing a specific amino acid substitution located in a B or T cell epitope may be a useful tool for examining the epitope.

## Materials and methods

### Needle and pollen DNA

Needles were sampled from 47 *Chamaecyparis obtusa* plus trees at the Sanmu (35.64152N/ 140.37328E) and Ohtaki (35.18267N/ 140.23231E) seed orchards of Chiba Prefectural Agriculture and Forestry Research Center with permission of Chiba Prefecture. Pollen collected from 18 *Chamaecyparis obtusa* trees grown in an artificial forest located in Chiba Prefecture was purchased (alpha-Pinene, Katori, Chiba, Japan). Collected pollen were mixed and stored at –20°C. *Chamaecyparis obtusa* pollen from Ibaraki Prefecture was purchased (Yamizo pollen study group, Kuji, Ibaraki, Japan). Pollen was examined by microscopy to confirm morphologically no contamination of pollen from other species. DNA was extracted from needles and pollen using the DNeasy-Plant Mini kit (Qiagen, Hilden, Germany). Pollen was grounded with a mortar and pestle, whereas needles were minced and homogenized (disposable generator, Hard Tissue Tip, TH, OMNI Inc., Kennesaw, GA, USA) before DNA extraction.

### Library preparation and next-generation sequencing (NGS)

Cha o 1 or Cha o 2 genomic DNA sequences were amplified by PCR using Gflex (Takara Bio Inc., Kusatsu, Shiga, Japan). PCR was performed by 40 cycles at 98°C for 10 s, 60°C for 15 s, and 68°C for 30 s with a thermal cycler. For Cha o 1, forward (5’-cctgtaccttattagcagtccttgttttcc-3’ or 5’-ctgctccctaaaaatggcttcc-3’) and reverse (5’-agatagtacgacattctatattttgtggatca-3’ or 5’-agtccaacaatatacatcaca-3’) primers were based on the Cha o 1 cDNA sequence (D45404) and used in PCR. For Cha o 2, forward (5’-tatcgaacaatatcttagatcgaatcggagttt-3’) and reverse (5’-tggatgaacaataattaactttggcttgcatgg-3’) primers were based on the Cha o 2 genomic DNA sequence (AB211843.1) and the Cha o 2 cDNA sequence (Mori *et al*., 1999; data have not been deposited in a public database), respectively. PCR products were purified using the QiaQUICK PCR purification kit (Qiagen). The sequencing library was prepared from mixed PCR products using the Nextera XT DNA Library Prep kit (Illumina, San Diego, CA, USA) with the Nextera XT DNA Library Index kit (Illumina). For individuals whose original clones had not been confirmed at the molecular level, needles from three stands for each ID were obtained and equal amounts of amplified PCR products from these individuals were pooled. If more than three alleles were detected by NGS, the two major alleles were considered as true alleles for the plus trees in this work. We obtained paired-end libraries that were pooled and run simultaneously on an Illumina Miseq sequencing system by using the Miseq reagent kit v3 of the 150 bp paired-end (Illumina). Nucleotide sequence data are available from the DDBJ Sequenced Read Archive under accession numbers DRX249994–DRX250043.

### Data analysis

Indexed sequences were de-multiplexed and analyzed individually. Read cleaning, mapping and variant calling were performed by Genedata Expressionist for Genomic Profiling ver. 9.1.4a (Genedata AG, Basel, Switzerland). Briefly, adaptors and low-quality reads were removed by Trimmomatic (Ver. 0.32) with the following parameters: ILLUMINACLIP: NexteraPE-PE.fa:2:30:10; LEADING: 10; TRAILING: 10; SLIDINGWINDOW: 4:15; and MINLEN: 36. Reads were mapped to the reference sequences of Cha o 1 (LC586088) or Cha o 2 (AB211843) by BWA-MEM (ver. 0.7.12) using the default settings. The reference sequence of Cha o 1 was obtained by Sanger sequencing of the PCR product from Miho 6 needle DNA, and both strands were determined. SNPs/Indels were detected by using the variant caller in Genedata Expressionist for Genomic Profiling (ver. 9.1.4a). Nonsynonymous SNPs were detected by SnpEff (ver. 3.6c) using the default settings.

### PCR-Restriction Fragment Length Polymorphism (RFLP)

A partial Cha o 1 genomic DNA sequence was amplified by PCR using Gflex (Takara Bio Inc.) and a pair of DNA primers with the following sequences: forward (5’-tgtagggcacgatatggactt-3’) and reverse (5’-tgcatgttagaaccccagca-3’). The primers were based on the Cha o 1 genomic DNA sequence (LC586088). Amplification was performed by 40 cycles at 98°C for 10 s, 60°C for 15 s, and 68°C for 30 s with a thermal cycler. The PCR product was purified using the QIAEX II Gel Extraction kit (Qiagen) and restricted by *Psi*I (NEB, Ipswich, MA, USA). The length of the restricted PCR product was examined by TapeStation and D1000 ScreenTape instruments (Agilent, Santa Clara, CA, US).

## Results

### Cha o 2 allergen polymorphisms in pollen

**Fig 1:**
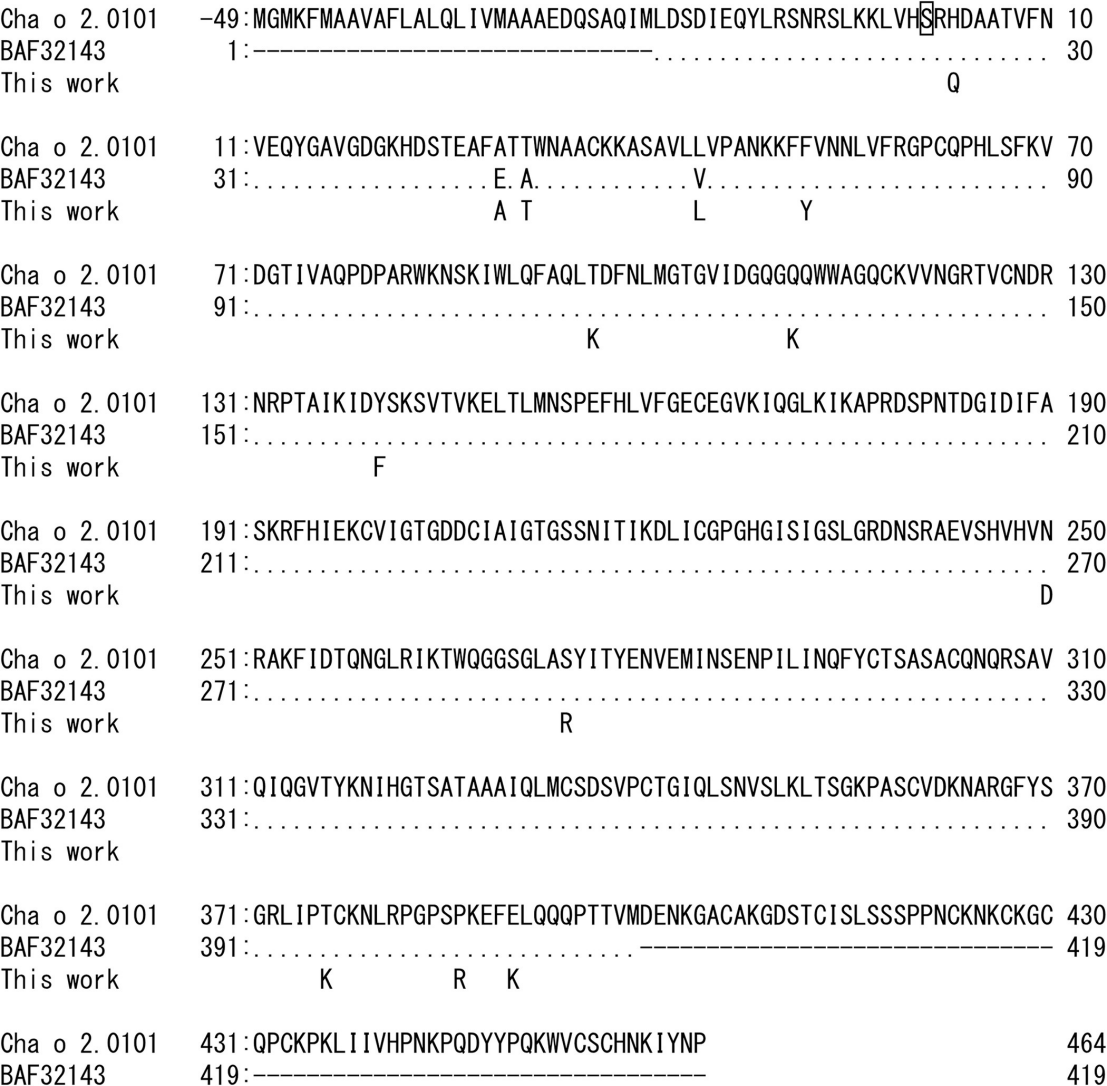
Comparison of amino acid sequences of isoallergen Cha o 2.0101 and those deduced from genomic sequence data of the Cha o 2 gene. The N-terminal amino acid of isoallergen Cha o 2.0101 after processing is boxed and numbered as 1 [15, 16]. The three residues that differed in BAF32143 [22] are indicated and other residues are shown as ‘.’. Undetermined residues are shown as ‘–‘. Substitutions discovered in this study are shown below the BAF32143 sequence.

**Fig 2:**
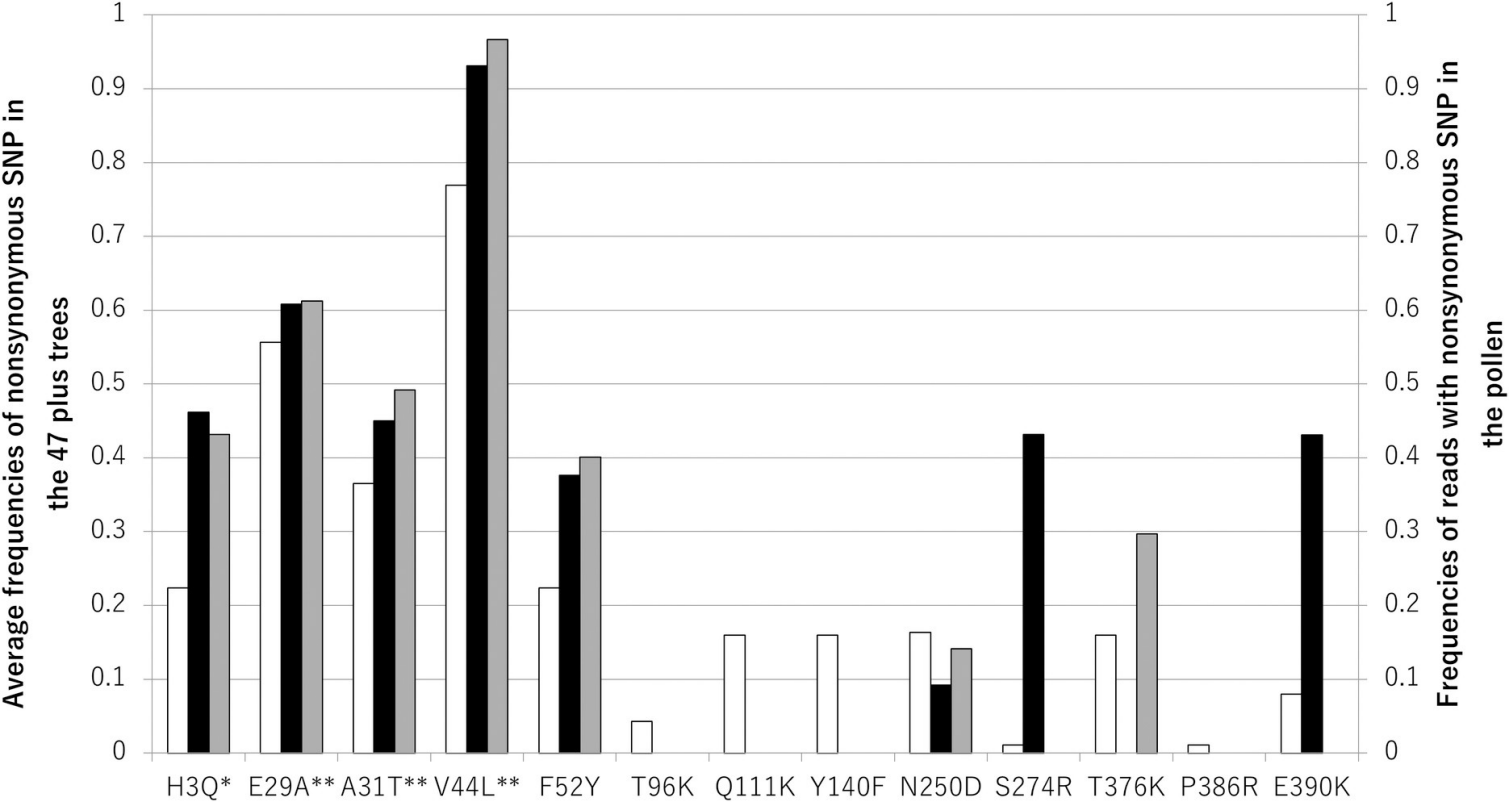
Comparison of frequencies for deduced polymorphic residues of Cha o 2 in plus trees from the seed orchards and those reads with nonsynonymous SNPs in Cha o 2 from pollen. Average frequencies of deduced polymorphic residues of Cha o 2 in the 47 plus trees of the seed orchards from Chiba Prefecture (open bars, left axis) were compared with those reads with nonsynonymous SNPs in Cha o 2 from pollen taken from the artificial forests in the same prefecture (closed bars, right axis) and Ibaraki Prefecture (shaded bars, right axis). *: H3Q was determined previously by N-terminal amino acid sequencing [16]. **: E29A, A31T and V44L were reported in [15] by the deduced amino acid sequence (Q7M1E7) from cDNA.

We explored Cha o 2 allergen polymorphism in pollen collected from artificial forests located in Chiba and Ibaraki prefectures. DNA sequences of Cha o 2 amplified from pollen DNA were examined by NGS. The genomic DNA sequence of Cha o 2 (AB211843.1) isolated from Uwajima 6 [22] was used as a reference, although the amino acid sequence (BAF32143) deduced from AB211843.1 differed by three amino acids when compared with that of isoallergen Cha o 2.0101 (Fig 1) [15, 24]. Pollen was collected from 18 trees in Chiba Prefecture. *Chamaecyparis obtusa* is diploid and Cha o 2 is probably a single copy gene. Thus, maximum theoretical number of isoforms possible in the Chiba pollen is 36 (i.e., 18 trees multiplied by 2). The number of trees used for collecting pollen from Ibaraki Prefecture was unknown. No nonsense mutations were present. Sequencing analysis of pollen collected from Chiba Prefecture revealed eight amino acid substitutions (H3Q, E29A, A31T, V44L, F52Y, N250D, S274R and E390K) in Cha o 2 whereas seven (H3Q, E29A, A31T, V44L, F52Y, N250D and T376K) amino acid substitutions in Cha o 2 were identified for pollen collected from Ibaraki Prefecture (Fig 2). Six (H3Q, E29A, A31T, V44L, F52Y and N250D) of these substitutions were found in pollen from both prefectures, and the frequencies of their reads were similar between the two sources (Fig 2). Information on haplotypes of Cha o 2 and the frequency of the isoforms were not obtained because our analysis used short reads of 150 bp.

### Cha o 2 DNA sequencing results from plus tree needles

Cypress trees located in artificial forests of Chiba Prefecture are probably progenies of plus trees kept in the seed orchards at Chiba Prefectural Agriculture and Forestry Research Center. To compare substitutions detected in pollen collected from Chiba Prefecture, Cha o 2 DNA sequences from plus trees kept in the seed orchards were examined. Needle DNA mutations in the Cha o 2 gene were surveyed by NGS for the 47 plus trees. Thirteen nonsynonymous SNPs of the Cha o 2 gene were identified. The amino acid substitutions deduced were H3Q, E29A, A31T, V44L, F52Y, T96K, Q121K, Y140F, N250D, S274R, T376K, P386R and E390K (Table 1), and their average frequencies in the 47 trees ranged between 0.01 and 0.77 (Fig 2). A no nonsense mutation was detected. All Cha o 2 substitutions deduced in pollen from Chiba and Ibaraki prefectures were also found in the plus trees. No plus tree was found to be homozygous for the Cha o 2 allele encoding isoallergen Cha o 2.0101. Possible heterozygous plus trees for the allele encoding Cha o 2.0101 were found (e.g., Kuno 2 in Table 1). Three plus trees, Miho 6, Naka 6 and Nishikawa 17, were deduced to express the Cha o 2 isoform of BAF32143. Of the 47 plus trees, 42 were found to be heterozygous for one or more of the nonsynonymous mutations in the Cha o 2 gene.

**Table 1:** Polymorphisms of Cha o 2 deduced by DNA sequencing of plus trees from the seed orchards of Chiba Prefecture.

	AA^a^												
Mother tree	3	29	31	44	52	96	111	140	250	274	376	386	390
Reference	H	E	A	V	F	T	Q	Y	N	S	T	P	E
(BAF32143)													
Frequency of substitution^b^	0.22	0.56	0.37	0.77	0.22	0.04	0.16	0.16	0.16	0.01	0.16	0.01	0.08
Miho 6	.	.	.	.	.	.	.	.	.	.	.	.	.
Naka 6													
Nishikawa 17													
Tanzawa 7	./Q	.	.	./L	./Y	.	.	.	./D	.	.	.	.
Shisui 1	./Q	.	.	./L	./Y	.	.	.	D	.	.	.	.
Chiba 6	Q	.	.	L	Y	.	.	.	./D	.	.	.	.
Tanzawa 8													
Kinada 4	./Q	./A	.	L	./Y	.	./K	./F	.	.	.	.	.
Kinada 5	./Q	./A	.	L	./Y	.	./K	./F	./D	.	.	.	.
Tanzawa 5													
Nishitama 1	./Q	./A	.	L	./Y	.	./K	./F	.	./R	.	.	./K
Chiba 5	./Q	./A	./T	L	./Y	.	./K	./F	./D	.	./K	.	./K
Nishikawa 14	./Q	./A	./T	./L	./Y	.	.	.	./D	.	.	.	./K
Nishikawa 6	./Q	./A	./T	./L	./Y	.	.	.	./D	.	./K	./R	./K
Hakone 3	./Q	./A	./T	L	./Y	.	.	.	./D	.	.	.	.
Kataura 4													
Naka 1	./Q	./A	./T	L	./Y	.	.	.	./D	.	./K	.	.
Nishikawa 5													
Nishitama 4	./Q	./A	./T	L	./Y	.	.	.	.	.	./K	.	./K
Shinshiro 2													
Kataura 3	./Q	./A	./T	L	./Y	.	.	.	.	.	.	.	./K
Satsugo 4	./Q	./A	./T	L	./Y	.	.	.	.	.	.	.	.
Chiba 3	.	./A	.	./L	.	./K	.	.	.	.	.	.	.
Naka 9													
Chiba 4	.	./A	.	./L	.	.	./K	./F	.	.	.	.	.
Nishitama 5	.	A	.	L	.	.	K	F	.	.	.	.	.
Hakone 5	.	./A	./T	./L	.	.	.	.	.	.	.	.	.
Kodama 3													
Nishikawa 11													
Satsugo 2													
Takahagi 3													
Hakone 1	.	A	./T	L	.	./K	.	.	.	.	./K	.	.
Kinada 7													
Chiba 2	.	A	./T	L	.	.	./K	./F	.	.	.	.	.
Hakone 4													
Kataura 5													
Kataura 6													
Nishikawa 18													
Nishitama 6													
Satsugo 3													
Kuno 2	.	A	T	L	.	.	.	.	./D	.	.	.	.
Tanzawa 6	.	A	T	L	.	.	.	.	./D	.	./K	.	.
Akimoto 1	.	./A	./T	./L	.	.	.	.	.	.	./K	.	.
Minamitama5													
Mito 1	.	A	T	L	.	.	.	.	.	.	K	.	.
Hakone 2	.	A	T	L	.	.	.	.	.	.	./K	.	./K
Tanzawa 2	.	.	.	.	.	.	.	.	.	.	./K	.	.
Cha o 2.0101	.	A	T	L	.	.	.	.	.	.	.	.	.

Only substituted residues from the reference are shown.

^a^Amino acid residues in the mature form of Cha o 2 are shown.

^b^Average frequencies of alleles encoding substitutions are shown.

–: without substitution;–/X: both the reference and subsituted residue ‘X’ were deduced; X: substitution was deduced.

### Cha o 1 allergen polymorphisms in pollen

We next explored Cha o 1 allergen polymorphism in pollen collected from artificial forests located in Chiba and Ibaraki prefectures. DNA sequences of Cha o 1 amplified from pollen DNA were determined by NGS. The genomic DNA sequence of Cha o 1 from the plus tree Miho 6 was sequenced by the Sanger method (LC586088) because no genomic DNA sequence of the Cha o 1 gene was available. The DNA sequence of the exons of LC586088 was found to be identical to that of cDNA reported previously (D45404) [14], and thus the reference sequence used in this report encodes the Cha o 1.0101 isoallergen. The same pollen DNA used for the survey of polymorphism in Cha o 2 was examined. No nonsense mutations were found. No non-synonymous mutation in Cha o 1 was identified in pollen from Chiba and Ibaraki prefectures.

### Cha o 1 DNA sequencing results from plus tree needles

**Fig 3:**
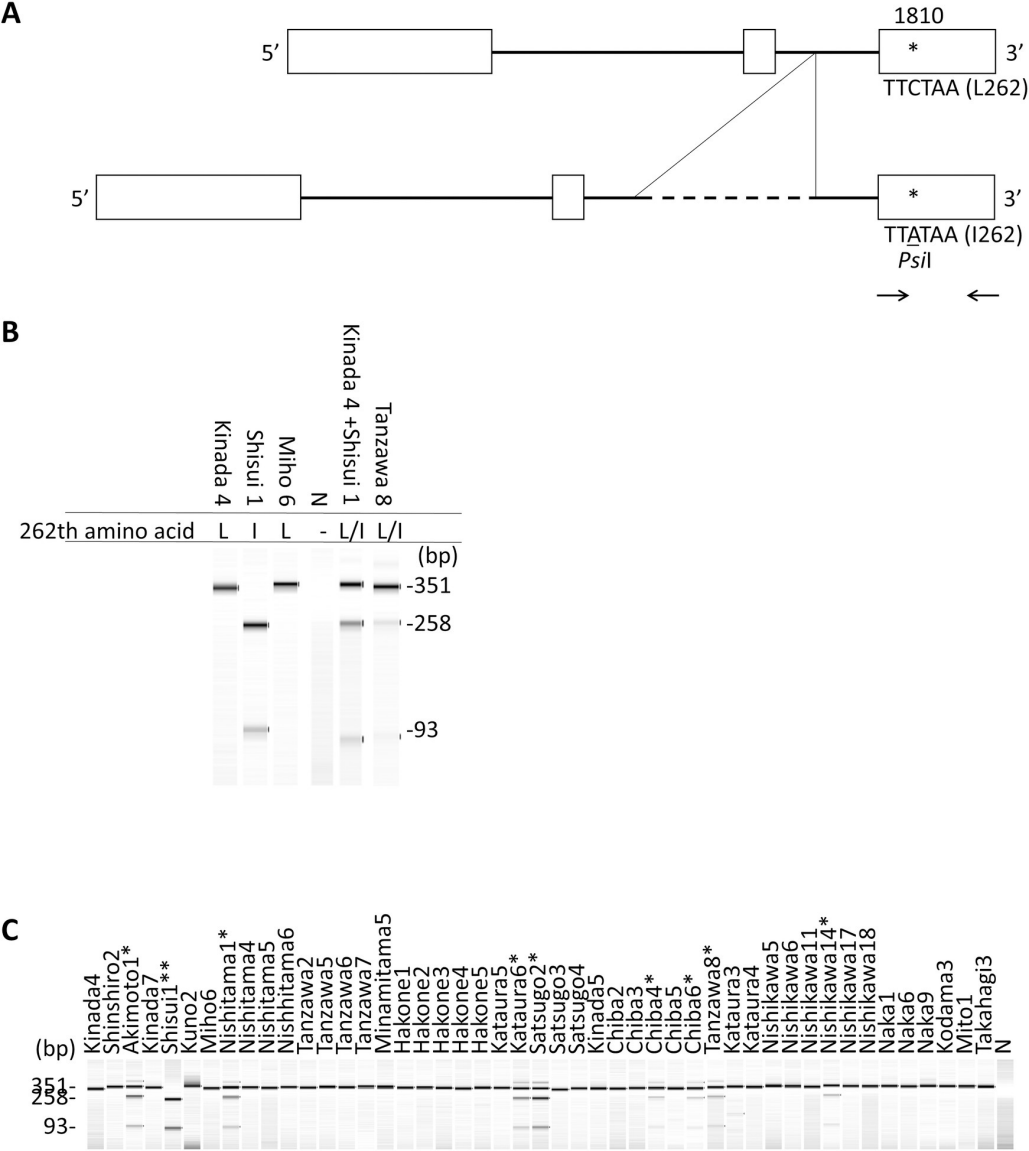
Cha o 1 gene organization. The genomic DNA of the Cha o 1 gene from the plus tree Miho 6 was amplified and sequenced (Genbank accession number #LC586088). (A) Comparison with the cDNA sequence of Cha o 1 reported previously (Genbank accession number #D45404) [14]. The exon and intron structures were determined. The DNA sequence for the exon of Cha o 1 from Miho 6 was identical to reported cDNA and was used as the reference sequence for NGS in this report. Cha o 1 genomic DNA of the Shisui 1 plus tree has a second intron that is ~0.6 kb longer and the 1810th nucleotide (asterisk) is mutated from cytosine to adenine, which gives rise to the L262I substitution. The newly formed SNP afforded the *Psi*I restriction site, TTATAA. Primer pairs for PCR amplification and detection of the SNP are shown as arrows. (B) Electrophoresis of the restricted PCR product by *Psi*I. PCR products of Kinada 4, Miho 6 were not restricted by *Psi*I, which indicates that residue 262 of them are Leucine. The PCR product from Shisui 1 was completely restricted, which indicates that residue 262 is Isoleucine. From a mixture of Kinada 4 and Shisui 1, an artificial heterozygote for the substitution was prepared and its PCR product was partially restricted. A similar band pattern was obtained from Tanzawa 8, which indicates that residue 262 was a mixture of Leu and Ile. (C) PCR-RFLP analysis of the 47 mother trees from the seed orchards of Chiba Prefecture. PCR products of the 3^rd^ exon of Cha o 1 were analyzed for the presence of the *Psi*I recognition site. Homozygotes (*) and heterozygotes (**) for the substitution of Cha o 1[L262I] are shown. N: negative control, PCR product without template treated with *Psi*I.

The DNA sequence of Cha o 1 was determined by NGS using needle samples from the 47 plus trees. NGS of the needle samples taken from the 47 plus trees detected only one substitution (L262I) in Cha o 1 from the plus tree Shisui 1. Shisui 1 is a possible homozygote for the non-synonymous mutation because no read without the mutation was detected. The Cha o 1 PCR product from Shisui 1 was 0.6 kb longer than those from other plus trees, which indicates the presence of a 0.6 kb insertion into the Cha o 1 gene from Shisui 1 (Fig 3A and 3B). This possible insertion is located in the 2nd intron because NGS analysis revealed a gap in the 2nd intron of the gene. A no nonsense mutation was detected in the 47 plus trees.

### PCR-RFLP of Cha o 1[L262I] from plus trees

We hypothesized that the SNP responsible for L262I in Cha o 1 was linked to the 0.6 kb insertion. In this case, the SNP would be difficult to detect from heterozygotes with the mutation by NGS because a longer sequence is generally not as efficiently amplified by PCR. A PCR-RFLP marker was developed to solve this issue, which detects both alleles with and without the mutation (Fig 3A, 3B). Shisui 1 was found to be homozygous for the mutation from this marker analysis, which is consistent with the results from NGS. The marker analysis confirmed that Miho 6, whose Cha o 1 sequence was identical to Cha o 1.0101, did not include the mutation. An artificial heterozygote for the mutation had both alleles; with and without the mutation (Fig 3B). The PCR-RFLP marker analysis revealed that 8 of the 47 trees in the orchards of Chiba Prefecture were heterozygotes for the SNP (Fig 3C, Table 2). The other 38 trees were found to not express the Cha o 1 with the L262I substitution. The L262I substitution was not detected in Cha o 1 sequences that were derived from pollen samples collected in Chiba and Ibaraki prefectures (data not shown).

**Table 2:** Polymorphisms of Cha o 1 for plus trees taken from the seed orchards of Chiba Prefecture determined by PCR-RFLP.

	AA
Plus tree	262
Reference*	L
Frequency of substitution**	0.11
Chiba 2, Chiba 3, Chiba 5, Hakone 1, Hakone 2, Hakone 3, Hakone 4, Hakone 5, Kataura 3, Kataura 4, Kataura 5, Kinada 4, Kinada 5, Kinada 7, Kodama 3, Kuno 2, Minamitama 5, Miho 6, Mito 1, Naka 1, Naka 6, Naka 9, Nishikawa 5, Nishikawa 6, Nishikawa 11, Nishikawa 17, Nishikawa 18, Nishitama 4, Nishitama 5, Nishitama 6, Satsugo 3, Satsugo 4, Shinshiro 2, Takahagi 3, Tanzawa 2, Tanzawa 5, Tanzawa 6, Tanzawa 7	.
Akimoto 1, Chiba 4, Chiba 6, Kataura 6, Nishikawa 14, Nishitama 1, Satsugo 2, Tanzawa 8	./I
Shisui 1	I

.: without substitution;–/I: both the reference and substituted residue ‘I’ were deduced; I: substitution was deduced.

*Amino acid residue in mature form of Cha o 1 is shown.

**Frequencies of substituted residues for the 47 plus trees are shown.

## Discussion

We identified a single amino acid substitution and 13 amino acid substitutions in Cha o 1 and Cha o 2 allergens, respectively, from pollen collected in Chiba and Ibaraki prefectures and from needles of 47 plus trees located at the seed orchards of Chiba Prefecture.

### Homogeneity of Cha o 1

DNA encoding Cha o 1 with the L262I substitution from the plus trees of seed orchards was not detected by NGS or PCR-RFLP in pollen DNA collected in the Chiba and Ibaraki prefectures. However, this result does not exclude the possibility that Cha o 1 from pollen has the L262I substitution. Amplification of the allele coding I262 was probably lower when compared with the amplification of the allele coding L262 because the allele coding I262 yielded a longer PCR product. The NGS and PCR-RFLP data suggested a possible link between the 0.6 kb insertion in the 2nd intron of the Cha o 1 gene and the non-synonymous mutation. The sensitivity of the PCR-RFLP method developed for detecting the L262I substitution may have been too weak to detect this mutation in DNA taken from the pollen. Amplicon sequencing of the 3rd exon of Cha o 1 used in PCR-RFLP should reveal the possible L262I mutation in pollen DNA from Japanese cypress in Chiba and Ibaraki prefectures.

We conclude that isoallergen Cha o 1.0101 is a major variant of Cha o 1 from artificial forests in Chiba Prefecture. The presence of the L262I substitution in Cha o 1 should have a limited effect on recognition by antibodies because Leu and Ile share the same molecular weight and hydrophobic properties [25, 26], and the average frequency of the allele among the 47 plus trees is only 0.11. Because no substitutions other than L262I were found in Cha o 1 from plus trees and pollen, Cha o 1 derived from pollen collected in Chiba Prefecture may behave uniformly when interacting with its antibodies that target Cha o 1.

### Heterogeneity of Cha o 2

In contrast to Cha o 1, we detected 13 amino acid substitutions in Cha o 2. Among the 47 plus trees, no homozygous tree for the allele encoding isoallergen Cha o 2.0101 was detected. Of the 47 plus trees, 42 were heterozygotes for one or more of the 13 substitutions. This indicates that purified Cha o 2 derived from pollen collected in Chiba Prefecture was a heterogeneous allergen based on amino acid sequence analysis. Information on haplotypes of Cha o 2 and their frequencies were not obtained because our analysis used short reads of 150 bp. Out of 13 substitutions, 7 were also detected in pollen collected from 18 trees located in artificial forests in Chiba Prefecture. Furthermore, the read frequencies derived from NGS of five (E29A, A31T, V44L, F52Y, N250D) substitutions found in pollen were similar to the corresponding read frequencies obtained for the 47 plus trees (Fig 2). This is consistent with trees from artificial forests in Chiba Prefecture being likely progenies of the 47 plus trees. Five of the remaining eight substitutions were not detected in pollen from Chiba Prefecture and the read frequencies of H3Q, S274R and E390K differed from the corresponding average frequencies determined for the 47 plus trees (Fig 2). This observation may arise because the collected pollen sample size was small. Thus, collecting and analyzing larger sample sizes should give frequencies that are similar to those observed for the 47 plus trees.

Eight substitutions were detected in pollen Cha o 2 collected from Ibaraki Prefecture, which is located just north of Chiba Prefecture. These eight substitutions are also observed as part of the 13 substitutions found for Cha o 2 of plus trees from seed orchards of Chiba Prefecture. Ibaraki and Chiba prefectures belong to the same breeding district. In the same breeding district, seeds and seedlings can be shared for plantation. Therefore, species identified with substitutions in Cha o 2 may be similar between prefectures of the same district. About 1,000 plus trees of *Chamaecyparis obtusa* were selected and their progenies were planted throughout Japan, except for Hokkaido and Okinawa. Analysis of allozyme variation in plus trees of *Chamaecyparis obtusa* in Japan showed that the genetic differences among the breeding districts were smaller to those within the districts [27]. In breeding districts other than Chiba Prefecture belongs to, amino acid substitutions were not surveyed in this report and form a future research endeavor.

### Significance of Cha o 2 residue substitutions

Cha o 2 was identified to have 13 substituted residues in its amino acid sequence and some trees had multiple polymorphisms on Cha o 2. Thus, the anti-Cha o 2 antibody may show different affinities toward Cha o 2 collected from different sites or different trees (Table 1). Studies requiring strict amino acid sequences, such as in the analysis of T cell epitope, may be affected by the pollen batch because a single amino acid substitution in the T cell epitope may affect T cell reactivity [28].

Our study provides useful data for developing an ELISA that quantifies Cha o 2 accurately. mAb may fail to recognize its antigen when a single amino acid substitution occurs in its epitope. For example, substitution at residues 55 and 352 of Cry j 1 was found to affect recognition by the monoclonal antibodies (mAbs) [20]. This situation potentially occurs for any allergen purified from natural products. Information on polymorphic amino acid residues in target allergens should facilitate appropriate antibody selection for robust ELISA development. Identification of non-synonymous mutations in advance should enable assessment of the effect of mutation on the affinity of mAbs by using pollen extracts derived from the plus trees. In this work we identified 13 amino acid substitutions in Cha o 2, which suggests that mAbs whose epitopes do not include these 13 residues should be ideally selected for developing ELISA to quantitate Cha o 2 levels.

Determination of the epitope sequence of the mAb is essential for allergy research. Usually synthetic peptide libraries based on the sequence of the isoallergen are used. However, in some cases, ambiguous results such as the presence of multiple positive peptides in distant regions prohibit accurate analysis. Substitutions in epitopes may help explain such cases. As Cha o 2 was found to have 13 substitutions and some of the substitution frequencies were significant, it is possible that an anti-Cha o 2 mAb raised against variants other than the isoallergen Cha o 2.0101 should be taken into consideration when an epitope is examined.

**Fig 4:**
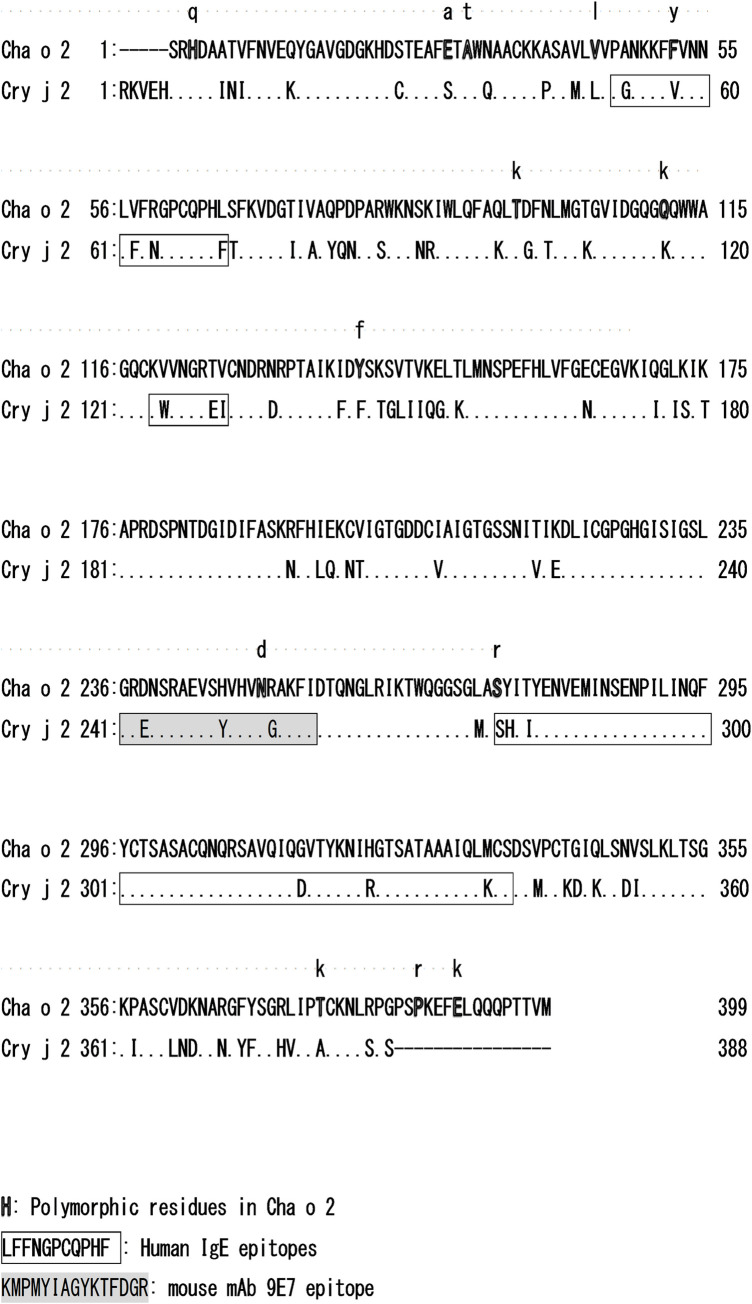
Comparison of the Cha o 2 and Cry j 2 amino acid sequences. Polymorphic residues in Cha o 2 (outlined letters), substituted residues (lower letters in the Cha o 2 sequence), IgE epitopes (boxed sequence), and the mouse mAb 9E7 epitope (grey shaded sequence) are shown.

Plus trees expressing Cha o 2 with substitutions may be useful for studying structural epitopes. Pollen from trees with the same mutation can be collected and Cha o 2 with the specific substitution may be purified. The two polymorphic residues F52 and S274 of Cha o 2 are included in the corresponding sequences to the IgE epitopes of Cry j 2 of Japanese cedar pollinosis patients (Fig 4) [29–31]. This raises the possibility that the affinity of IgE toward Cha o 2 is dependent on residues 52 and 274. Synthetic peptides are often used for examining sequential epitopes, although this is not always suitable for structural epitopes [29]. For examining structural epitopes of Cha o 2, isoforms of Cha o 2 may become useful tools to characterize structural epitopes. In the present study, we did not examine the effect of substitutions on affinity to IgE from Japanese cedar pollinosis patients. To examine this, purified Cha o 2 with or without the substitution is necessary. Purifying an isoform of Cha o 2 with F52Y or S274R is difficult at present because (1) purification of Cha o 2 requires a considerable amount of pollen material, which is difficult to obtain because the number of plus trees with mutations encoding F52Y and S274R was only two and one, respectively (Table 1), and (2) a non-synonymous mutation encoding S274R was found only as a heterozygote (Nishitama 1). Further surveying of polymorphisms in Cha o 2 of plus trees will be helpful because homozygotes for specific non-synonymous mutations may be detected and multiple trees with the same non-synonymous mutation may be found.

## Conclusion

We found 1 and 13 amino acid substitutions in Cha o 1 and Cha o 2, respectively, from plus trees located at nurseries in Chiba Prefecture and pollen collected in Chiba and Ibaraki prefectures. These polymorphisms in Cha o 2 may affect mAb affinity toward Cha o 2 when the antibody against Cha o 2 has been raised against a particular Cha o 2 amino acid sequence where one or more of the 13 substitutions resides in the antibody epitope.
